# Simultaneously Constructing Active Sites and Regulating Mn–O Strength of Ru‐Substituted Perovskite for Efficient Oxidation and Hydrolysis Oxidation of Chlorobenzene

**DOI:** 10.1002/advs.202205054

**Published:** 2022-11-27

**Authors:** Xiaoxiao Duan, Ting Zhao, Ben Niu, Zheng Wei, Ganggang Li, Zhongshen Zhang, Jie Cheng, Zhengping Hao

**Affiliations:** ^1^ National Engineering Laboratory for VOCs Pollution Control Material & Technology Research Center for Environmental Material and Pollution Control Technology University of Chinese Academy of Sciences Beijing 101408 P. R. China

**Keywords:** active sites, chlorinated volatile organic compounds (CVOCs) elimination, hydrolysis oxidation, Mn–O bond strength, perovskite oxides

## Abstract

Chlorinated volatile organic compounds (CVOCs) are a class of hazardous pollutants that severely threaten environmental safety and human health. Although the catalytic oxidation technique for CVOCs elimination is effective, enhancing the catalytic efficiency and simultaneously inhibiting the production of organic byproducts is still of great challenge. Herein, Ru‐substituted LaMn(Ru)O_3+_ *_*δ*_* perovskite with Ru–O–Mn structure and weakened Mn–O bond strength has been developed for catalytic oxidation of chlorobenzene (CB). The formed Ru–O–Mn structure serves as favorable sites for CB adsorption and activation, while the weakening of Mn–O bond strength facilitates the formation of active oxygen species and improves oxygen mobility and catalyst reducibility. Therefore, LaMn(Ru)O_3+_ *_*δ*_* exhibits superior low‐temperature activity with the temperature of 90% CB conversion decreasing by over 90 °C compared with pristine perovskite, and the deep oxidation of chlorinated byproducts produced in low temperature is also accelerated. Furthermore, the introduction of water vapor into reaction system triggers the process of hydrolysis oxidation that promotes CB destruction and inhibits the generation of chlorinated byproducts, due to the higher‐activity *OOH species generated from the dissociated H_2_O reacting with adsorbed oxygen. This work can provide a unique, high‐efficiency, and facile strategy for CVOCs degradation and environmental improvement.

## Graphical abstract

Constructing LaMn(Ru)O_3+_ *_*δ*_* perovskite with active sites (Ru–O–Mn) and weakened Mn–O bond strength facilitates chlorobenzene (CB) adsorption/activation and simultaneously improves the generation and mobility of active oxygen species, resulting in the higher efficiency of CB catalytic oxidation and more facile conversion of chlorinated byproducts. Hydrolysis oxidation further optimizes the process of CB degradation, promoting CB destruction, and inhibiting byproducts generation.

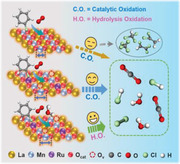

## 1 Introduction

As a kind of detrimental air pollutants with high toxicity, superior difficulty for biodegradation, and strong capacity for inducing secondary pollution (e.g., photochemical smog), chlorinated volatile organic compounds (CVOCs) pose a great threat to atmospheric environment and human health.^[^ ^1^, ^2^ ^]^ Due to the high purification efficiency and low energy consumption, catalytic oxidation has been accepted as a promising approach for CVOCs elimination, where these chlorinated pollutants are decomposed into inorganic products (CO_2_, H_2_O, and HCl/Cl_2_).^[^ ^3^, ^4^, ^5^ ^]^ Lowering the reaction temperature and synchronously inhibiting the generation of chlorinated byproducts is the research emphasis of CVOCs catalytic oxidation,^[^ ^6^ ^]^ and developing high‐efficiency and durable catalysts as well as optimizing the reaction process are practicable strategies to achieve the target.

Among numerous catalytic materials, Mn‐based perovskites with ABO_3_ structure (such as LaMnO_3_) are effective and inexpensive for CVOCs degradation.^[^ ^7^, ^8^ ^]^ However, the catalytic efficiency of Mn‐based perovskites need to be improved, and the problem of Cl poisoning, which usually causes catalyst deactivation and the formation of polychlorinated byproducts (that could be more harmful than origin pollutants), is also necessary to be overcome. In order to enhance the catalytic performance, boosting the ability of CVOCs adsorption/activation while promoting the active oxygen species generation, improving the oxygen mobility and catalysts reducibility is a desirable strategy. Recently, combining noble metals (NM) with transition metal oxides (TMO) is feasible to facilitate the adsorption and activation of reactants with the construction of NM–O–TM structure, due to that the electronic structure of metals could be adjusted through NM–O–TM sites and the interaction of reactants and metal sites are enhanced consequently.^[^ ^9^, ^10^, ^11^ ^]^ As for improving the properties of oxygen species and reducibility of perovskites, regulating B‐site metal–oxygen (M–O) bond strength could be an effective way, because the weakened metal‒oxygen bond strength is observed to enhance the physicochemical features (such as reducibility and surface oxygen species activity) of TMO catalysts.^[^ ^12^, ^13^ ^]^ Besides the doping of transition metals,^[^ ^14^ ^]^ the introduction of noble metals are also capable of weakening M–O bond strength and enhancing the catalytic performances of perovskites.^[^ ^15^, ^16^ ^]^ Among multifarious noble metals, Ru could accelerate the removal of Cl species and lessen the generation of chlorinated byproducts, making Ru‐containing materials suitable for CVOCs oxidation.^[^ ^17^, ^18^, ^19^, ^20^ ^]^ Inspired by the aforementioned results, the incorporation of Ru into Mn‐based perovskite has potential to promote the activation of CVOCs molecules and simultaneously enhance the properties of oxygen species and catalysts reducibility. Nevertheless, the structure–activity relationship of Ru‐containing perovskites is unclear and needs deep exploration for CVOCs oxidation.

Regulating the reaction process is also important for CVOCs decomposition, and the introduction of H_2_O, as a simple operation, would affect the process significantly.^[^ ^21^ ^]^ A hydrolytic destruction route was reported in chlorobenzene (CB) oxidation with the participation of H_2_O, which strengthened the catalysts stability and restrained the generation of polychlorinated byproducts.^[^ ^7^ ^]^ Our previous research also found that H_2_O and oxygen could be used as oxidant simultaneously for CVOCs destruction, which was defined as hydrolysis oxidation reaction.^[^ ^22^ ^]^ These results indicate H_2_O is a significant reaction agent in CVOCs oxidation, and hydrolysis oxidation could be a convenient way of optimizing the reaction process.

Enlighted by these researches, we propose a strategy of partial substitution of Ru into LaMnO_3+_ *_*δ*_* bulk to simultaneously construct active sites and regulate Mn–O bond strength for CB (a representative CVOCs) catalytic destruction. Based on systematical investigation by experimental and theoretical techniques, it is demonstrated the formed Ru–O–Mn sites facilitate CB activation, while the weakened Mn–O strength boosts the generation and mobility of active oxygen species and enhances the catalyst reducibility. Consequently, the oxidation of CB is remarkably improved (the reaction temperature is decreased by over 90 °C) and the deep conversion of chlorinated byproducts is more facile. Moreover, the introduction of H_2_O triggers hydrolysis oxidation of CB, which further promotes CB decomposition and inhibits chlorinated byproducts production, benefiting from the generated higher‐activity *OOH species that are revealed by reaction mechanism analyses. We believe this work can guide the design of efficient catalysts and provide advisable techniques (especially hydrolysis oxidation) for CVOCs elimination.

## 2 Results and Discussion

### 2.1 Characterizations of the Synthesized Catalysts

**Figure 1:**
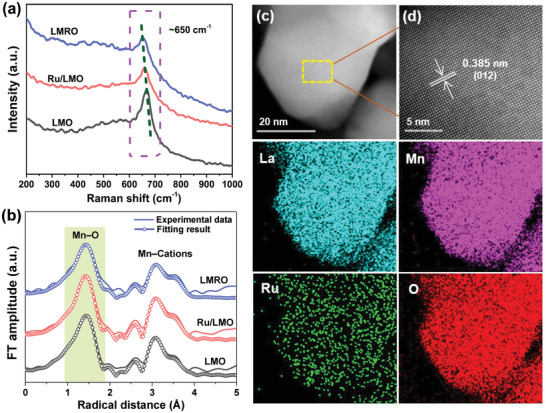
Structure characterizations. a) Ex situ Raman spectra and b) Mn K‐edge FT EXAFS spectra of LMRO, Ru/LMO, and LMO; c,d) AC‐HADDF‐STEM images and elements mapping of LMRO.

The X‐ray diffraction (XRD) patterns of catalysts (Figure S1a, Supporting Information) show well‐resolved diffraction peaks of standard perovskite structure (JCPDS No. 50–0298) with hexagonal phase (R‐3c space group). The lattice parameters (Table S1, Supporting Information) obtained from refined XRD patterns (Figure S2, Supporting Information) are similar for LMRO, Ru/LMO, and LMO, indicating that the introduction of Ru (bulk‐substituted and surface‐loaded) makes negligible difference in unit cell structure of perovskite. The Ru content of LMRO and Ru/LMO are 0.62% and 0.61%, respectively (Table S1, Supporting Information). Due to the high dispersion of Ru, no phases ascribed to ruthenium species are detected on LMRO and Ru/LMO. Transmission electron microscope (TEM) images show that RuO_2_ nanoparticles are supported on the surface of Ru/LMO but analogous RuO*_x_* species cannot be found on LMRO (Figure S3, Supporting Information). The results of high‐angle annular dark‐field scanning transmission electron microscope (HADDF‐STEM) indicate the (012) plane is mainly exposed for LMRO and further confirms the inexistence of RuO*_x_* nanoparticles or clusters (**Figure** **1**c,d). Besides, energy dispersive spectroscopy (EDS) mapping demonstrates a homogeneous distribution of La, Mn, O, and Ru in LMRO, which confirms that Ru is introduced into perovskite bulk, scilicet, the partial substitution of Ru for Mn in the lattice is realized. The homogeneous dispersed structure is beneficial for the interaction between Mn and Ru, and abundant Ru–O–Mn sites could be formed in LMRO. Raman spectroscopy are performed to investigate the surface structure of catalysts (Figure 1a). The dominant peak at ca. 650 cm^−1^ is assigned to Mn–O stretching vibration.^[^ ^23^ ^]^ After the introduction of Ru, Raman peaks of LMRO and Ru/LMO are red‐shifted compared with LMO, suggesting the Mn–O bond strength has been changed. According to Hooke's law, the Mn–O bond force constant (*k*) representing Mn–O bond strength is calculated via the following formula

(1) $$\omega =\frac{\sqrt{k/\mu }}{2\pi c}$$

where *ω* is the Raman shift, µ is effective mass of Mn–O bond and c is velocity of light. The calculation result manifests that Mn–O bond force constant of three catalysts is in the sequence of LMRO < Ru/LMO < LMO (Figure S4a, Supporting Information), demonstrating that Mn–O bond strength of perovskite has been weakened by the incorporation of Ru and this effect is more significant for partial substitution of Ru into bulk, which could be on account of the formation of more Ru–O–Mn structure.^[^ ^2^ ^]^ Additionally, Fourier‐filtered and Fourier transformed (FT) extended X‐ray absorption fine spectra (EXAFS) at Mn K‐edge are provided in Figure 1b and Figure S4b in the Supporting Information. For FT EXAFS profiles, the peak of first shell at around 1.5 Å is attributed to the Mn–O bond, while the peak of first shell (near 3.1 Å) should be assigned to the bond of Mn with the nearest cations (La and Mn).^[^ ^24^ ^]^ Based on the fitting results, Mn–O bond distances in LMRO, Ru/LMO, and LMO are 1.96, 1.94, and 1.91 Å, respectively (Table S2, Supporting Information), which further demonstrates that the Mn–O bond strength of catalysts follow the sequence of LMRO < Ru/LMO < LMO.

**Figure 2:**
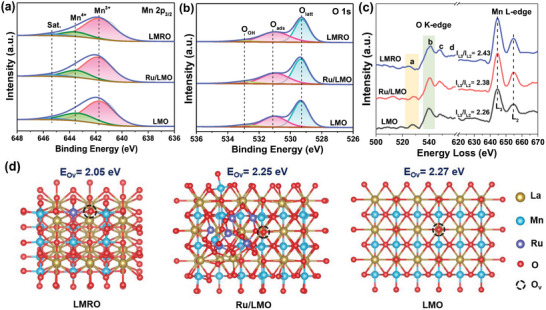
Characterizations of chemical states. a) Mn 2p_3/2_, b) O 1s XPS spectra, and c) EELS spectra with O K‐edge and Mn L‐edge of catalysts; d) Calculated structures of oxygen vacancy on LMRO, Ru/LMO and LMO.

The chemical states of surface element on catalysts are analyzed by X‐ray photoelectron spectroscopy (XPS). The XPS survey and La 3d spectra of the prepared catalysts are provided in Figure S5a,b in the Supporting Information. The Mn 2p_3/2_ spectra (**Figure** **2**a) display that the peaks of Mn^3+^ and Mn^4+^ are at the binding energy (BE) of 641.7 and 643.5 eV, respectively,^[^ ^8^, ^25^ ^]^ and a characteristic satellite peak of Mn^3+^ appears at 645.4 eV. The surface ratio of Mn^3+^/Mn^4+^ on LMRO (5.09) is higher than that on Ru/LMO (3.59) and LMO (2.72), implying the relative content of Mn^3+^ on LMRO is the highest. Furthermore, average oxidation state (AOS) of Mn cations is calculated based on Mn 3s spectra (Figure S5c, Supporting Information), which decreases as LMRO (3.37) < Ru/LMO (3.52) < LMO (3.56), consistent with their surface ratio of Mn^3+^/Mn^4+^. Hence, it can be found the incorporation of Ru into perovskite lattice results in LMRO owning the highest Mn^3+^/Mn^4+^ ratio and lowest Mn valence. It is generally accepted that higher Mn^3+^/Mn^4+^ ratio is associated with larger amounts of oxygen vacancy (O_v_),^[^ ^26^ ^]^ where molecular oxygen can be activated into reactive oxygen species for CB decomposition. For Ru species, the two peaks at around 464.2 and 486.4 eV in Ru 3p spectra (Figure S5d, Supporting Information) are correlated with 3p_3/2_ and 3p_1/2_ states of Ru^4+^,^[^ ^27^ ^]^ confirming the RuO_2_ on Ru/LMO surface and Ru^4+^ cations in LMRO. The surface contents of Ru/Mn for LMRO and Ru/LMO are about 2.1% and 2.7%, respectively. This ratio for LMRO is close to the theoretical value (circa 2.0%), while the higher ratio for Ru/LMO is due to the supported RuO_2_ are almost on the surface of perovskite support. In Figure 2b, the O 1s spectra are deconvoluted into three peaks at 529.3, 531.1, and 533.0 eV, which are attributed to surface lattice oxygen (O_latt_), surface adsorbed oxygen (O_ads_), and oxygen in surface adsorbed water (O_OH_), respectively.^[^ ^28^, ^29^ ^]^ Generally, O_ads_ composed of O_2_^−^, O_2_^2−^ and O^−^ are more easily to attack the organic molecules because of high electron density, showing high activity in oxidation of VOCs.^[^ ^30^ ^]^ Through normalized calculation of peak area (Table S3, Supporting Information), the O_ads_ concentration of LMRO (O_ads_/O_latt_ = 0.88) is higher than that of Ru/LMO (O_ads_/O_latt_ = 0.78) and LMO (O_ads_/O_latt_ = 0.64), suggesting the substitution of Ru is more conducive to the formation of O_ads_ compared to supporting Ru on the surface. Since gas‐phase O_2_ tend to be adsorbed on O_v_ to generate active O_ads_,^[^ ^31^ ^]^ the larger amount of O_ads_ means more O_v_ exist on LMRO, which is consistent with the analysis of Mn XPS spectra. Associated with foregoing Raman results, the weaker Mn–O strength of LMRO should be the reason of more O_v_ on it.

The coordination environment of the catalysts is further investigated by electron energy loss spectroscopy (EELS), which could characterize electronic properties and occupied states of orbitals.^[^ ^32^, ^33^, ^34^ ^]^ At the region of O K‐edge in Figure 2c, lower value of *I*_a_/*I*_b_ (intensity of peak (a) and (b)) indicates more O_v_ on the catalysts.^[^ ^35^ ^]^ According to the calculation results, *I*_a_/*I*_b_ decreases in the order of LMO (0.17) > Ru/LMO (0.16) > LMRO (0.14), signifying that LMRO possesses more O_v_. Two white lines in Mn L_2,3_‐edge are related to the transitions of excited‐state Mn to higher‐energy orbitals.^[^ ^34^ ^]^ Higher ratio of *I*_L3_/*I*_L2_ is associated with the lower valence of Mn, thus the sequence of *I*_L3_/*I*_L2_ (LMO < Ru/LMO < LMRO) suggests LMRO has the lowest valence of Mn. Actually, the formation of O_v_ will cause the reduction of Mn valence due to electrostatic balance. Therefore, besides XPS analyses, this result provides another evidence that more O_v_ are inclined to be formed on LMRO. Electron paramagnetic resonance (EPR) is also used for determining O_v_ (Figure S6, Supporting Information). The intensity of peak at *g* = 2.003 (represents O_v_) declines as LMRO > Ru/LMO > LMO, validating a larger content of O_v_ on LMRO. Moreover, density functional theory (DFT) calculations illustrate that the O_v_ formation energy (*E*_Ov_) of LMRO, Ru/LMO and LMO is 2.05, 2.25, and 2.27 eV, respectively (Figure 2d), suggesting the facile formation of O_v_ on LMRO. The existence of O_v_ brings about O_ads_, which could serve as active oxygen species for CB oxidation.^[^ ^8^, ^36^ ^]^

**Figure 3:**
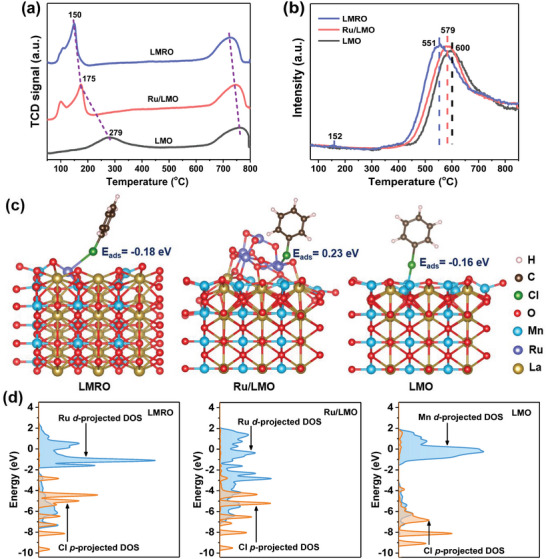
Identification of chemical properties and CB adsorption/activation. a) H_2_‐TPR and b) O_2_‐TPD of the prepared catalysts; c) structures of CB adsorbed on the surface of the perovskite‐based catalysts and d) the corresponding projected DOS.

The reducibility of catalysts is evaluated by H_2_ temperature‐programmed reduction (H_2_‐TPR). As illustrated in **Figure** **3**a, the peak centered at 145–400 °C is attributed to the reduction of Mn^4+^ to Mn^3+^, whereas the high‐temperature peak over 650 °C is associated with the reduction of Mn^3+^ to Mn^2+^.^[^ ^37^ ^]^ Besides these two major peaks, LMRO and Ru/LMO have another shoulder peak (90–145 °C) corresponding to the reduction of oxidized ruthenium. Compared with LMO, the reduction of Mn^4+^ are promoted by the introduction of Ru and the order of reduction temperatures is LMRO (150 °C) < Ru/LMO (175 °C) < LMO (279 °C). Such promotion should derive from the hydrogen‐spillover effect of metallic Ru and the weakened Mn–O bond strength.^[^ ^2^ ^]^ In fact, LMRO inclines to have weaker H_2_‐spillover effect than Ru/LMO, because most Ru substituted in bulk are difficult to be reduced or move to surface.^[^ ^37^ ^]^ However, the reduction of Mn^4+^ on LMRO is more facile than that on Ru/LMO, and the reduction temperature of Mn^3+^ on LMRO is also the lowest among the catalysts. These further demonstrate that the substitution of Ru weakens Mn–O bond strength more effectively, resulting in LMRO performing better reducibility that is in favor of CB oxidation.^[^ ^38^ ^]^

O_2_ temperature‐programmed desorption (O_2_‐TPD) can provide dependable information about the mobility of oxygen species (Figure 3b). The major desorption peak between 450 and 750 °C is assigned to the desorption of O_latt_. The introduction of Ru distinctly decreases O_latt_ desorption temperature, while that of LMRO is lower than Ru/LMO, implying the O_latt_ on LMRO are easier to be released. Moreover, a weak peak attributed to O_ads_ desorption appears at 120–180 °C on LMRO, which cannot be detected on Ru/LMO or LMO. These results evidence that the mobility of O_ads_ and O_latt_ of LMRO are both higher, owing to the weaker Mn–O bond strength originating from Ru substitution. Additionally, the profiles of CO_2_ production in CB‐TPSR are displayed in Figure S7 in the Supporting Information. The peak at 50–250 °C is ascribed to the reaction between adsorbed CB with O_ads_, while another one at over 400 °C is due to the oxidation of CB by O_latt_. Both two peaks over LMRO are at much lower temperatures in comparison with Ru/LMO and LMO (the undetected peak on LMO over 400 °C indicates its inert O_latt_), suggesting the better oxidation ability and higher mobility of O_ads_ and O_latt_ on LMRO, which further verifies the conclusion of O_2_‐TPD. In situ Raman measurement is conducted to further study the dynamic evolution of structure and oxygen species of LMRO (Figure S8a, Supporting Information). With the increase of temperature, the peak of Mn–O bond is gradually red shifted, indicating the weakening of Mn–O bond strength. That is, Mn–O bonds of LMRO are more facile to be broken at higher temperature, thus abundant O_v_ could be formed and the mobility of lattice oxygen would be promoted, which generally improve the catalytic oxidation of VOCs.^[^ ^39^ ^]^ This is proved by the results in Figure S8b (Supporting Information), where the reaction rate of CB oxidation is negatively related to the Mn–O force constant, suggesting that weaker Mn–O bond strength causes higher reaction rate.

Pyridine‐IR spectra are recorded for exploring surface acidity and the detailed results are presented in Figure S9 and Table S4 in the Supporting Information. In general, Lewis acid sites are predominate on three catalysts. Compared with LMO, the Lewis acidity of Ru/LMO and LMRO gets lower, possibly due to the decrease of surface Mn^4+^ (Table S3, Supporting Information) after Ru introduction (the Lewis acid strength of Mn^4+^ is higher than that of Mn^3+^). Notably, Brønsted acid sites are not detected on LMO or Ru/LMO, but the signal of that is recorded on LMRO, and it is known that the existence of Brønsted acid sites is good for promoting dichlorination and enhancing the HCl selectivity.^[^ ^17^ ^]^

### 2.2 CB Adsorption/Activation over the Catalysts

The adsorption properties of CB are studied by CB temperature‐programmed desorption (CB‐TPD). No apparent desorption peaks can be observed on LMO and Ru/LMO, while the desorption of preadsorbed CB from LMRO can be obviously detected at 60–120 °C (Figure S10, Supporting Information), confirming the greater capacity of LMRO for CB adsorption. DFT simulations are performed to further explore the CB adsorption and activation over the catalysts. The structures of three catalysts with adsorbed CB are displayed in Figure 3c, where the CB adsorption energy (*E*_ads_) of LMRO (−0.18 eV) is higher than that of Ru/LMO (0.23 eV) and LMO (−0.16 eV). The higher *E*_ads_ allows LMRO to adsorb CB more easily on the substituted Ru sites of Ru–O–Mn structure. The adsorbed CB would be activated and dissociated with the breakage of C–Cl bonds, followed with being attacked by active oxygen species to accomplish the total decomposition. Based on the results of projected density of states (PDOS) after CB adsorption (Figure 3d), the hybridization band of Ru 4d and Cl 2p orbits is at the energy level of −8 to −6 eV on LMRO, lower than that on LMO (−7 to −5 eV). For Ru/LMO, the occupied Ru 4d does not match well to Cl 2p orbit, presenting a weaker adsorption of CB. The computational results further demonstrate that LMRO is more capable of adsorbing and activating CB molecules due to the abundant Ru–O–Mn active sites, which are crucial for enhancing CB complete oxidation.

### 2.3 Catalytic Performances for CB Oxidation and Hydrolysis Oxidation

**Figure 4:**
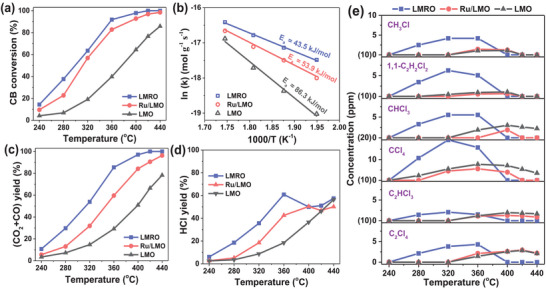
Catalytic performances of the catalysts for CB oxidation. a) Catalytic activity profiles; b) Arrhenius plots; c) CO*_x_* yield; d) HCl yield and e) distribution of chlorinated byproducts over as‐prepared catalysts during CB oxidation.

The activities of as‐prepared catalysts for CB oxidation are presented in **Figure** **4**a and the temperatures corresponding to 50% and 90% CB conversion (denoted as *T*_50_ and *T*_90_) are summarized in Table S3 in the Supporting Information. LMO shows a certain activity and the CB conversion reaches 90% over 440 °C. After the introduction of Ru, the conversion curve shifts towards low temperature, with *T*_50_ (298 °C) and *T*_90_ (356 °C) of LMRO much lower than that of Ru/LMO (*T*_50_ = 312 °C, *T*_90_ = 388 °C). Therefore, it can be found the activity of catalysts increases as LMO < Ru/LMO < LMRO, and the activity of LMRO surpasses many previously reported catalysts (Table S5, Supporting Information). The apparent activation energy (*E*_a_) is calculated with the elimination of kinetic mass transfer limitation and exhibited in Figure 4b. The *E*_a_ values of as‐prepared catalysts are in the reverse order of catalytic activity: LMO (*E*_a_ = 86.3 kJ mol^−1^) > Ru/LMO (*E*_a_ = 53.9 kJ mol^−1^) > LMRO (*E*_a_ = 43.5 kJ mol^−1^), which further confirms that partial substitution of Ru into bulk is better than supporting Ru on the surface of perovskite for improving reactants activation and CB degradation.

Figure 4c shows the yield of CO*_x_* (CO_2_ and CO) over all catalysts during the CB oxidation. The overall trend and order of CO*_x_* yield are similar to CB conversion and LMRO also has higher yield of CO*_x_* than other catalysts. At high temperature (above 420 °C), the yield of CO*_x_* (over 99%) is close to the conversion of CB on LMRO. But for Ru/LMO and LMO, the CO*_x_* yield is obviously lower than CB conversion, due to the slow conversion of intermediates on these two catalysts. These results demonstrate that LMRO possesses the stronger capacity of decomposing CB into inorganic CO*_x_*. As for the produced HCl, Figure 4d displays that the yield of HCl over LMO increases with the rise of temperature, due to the continuous conversion of CB. Differently, a decline could be noticed over LMRO and Ru/LMO, resulting from the oxidation of chlorine/HCl into Cl_2_, and this process would be facilitated at high temperature via Deacon reaction.^[^ ^17^ ^]^ Although raising temperature is in favor of Cl_2_ production, more CB would also be decomposed into HCl, thus the production of HCl increases again after the decline over LMRO and Ru/LMO. On the whole, the HCl yield over LMRO is the highest among three catalysts, due to its Brønsted acid sites and effective conversion of CB into HCl. During the reaction, HCl yield is lower than CB conversion over the prepared catalysts, owing to that partial chlorine is turned into Cl_2_, chlorinated byproducts and/or deposited on the surface of catalysts.

During the catalytic oxidation of CB, a few chlorinated organic byproducts are monitored including monochloromethane (CH_3_Cl), 1,1‐dichloroethylene (1,1‐C_2_H_2_Cl_2_), trichloromethane (CHCl_3_), tetrachloromethane (CCl_4_), trichloroethylene (C_2_HCl_3_), and perchloroethylene (C_2_Cl_4_), the evolution of which as a function of temperature are shown in Figure 4e. It has to be noted that no polychlorinated benzenes are detected under the detecting condition of gas chromatograph, which is quite different from the results of other reports.^[^ ^22^, ^38^ ^]^ The concentrations of the formed byproducts increase first and then decline along with the rise of reaction temperature. The maximum concentrations of these byproducts are lower than 10 ppm except for CCl_4_ (close to 20 ppm). As known, the generated C1 and C2 chlorinated organics come from the cracking of aromatic ring and chlorination by dissociated Cl species, which would be converted into CO_2_, H_2_O, and HCl/Cl_2_ when the temperature rises up. It can be seen LMRO affords more byproducts than Ru/LMO and LMO, and the generation of these by‐products over LMRO is at lower temperature. Higher concentration of byproducts occurring at lower temperature indicates LMRO possesses better reducibility and activity to decompose CB into various intermediates. These generated byproducts cannot be detected over LMRO when the temperature reaches 400 °C, due to the deep oxidation into final inorganic molecules. Nevertheless, although the other four byproducts disappear at 420 °C, C_2_HCl_3_ and C_2_Cl_4_ still can be monitored over Ru/LMO. As for LMO, the formed byproducts are difficult to be completely destructed, so CHCl_3_, CCl_4_, C_2_HCl_3_, and C_2_Cl_4_ all exist in outlet gas even at 440 °C. These phenomena confirm that LMRO has preferable ability of CB decomposition and deep oxidation of generated intermediates, which is consistent with the results of catalytic activity.

The long‐term stability of perovskite‐based catalysts are detected at 400 °C. As presented in Figure S11 (Supporting Information), all these catalysts basically maintain the high catalytic activity while a slight deactivation is observed, which could be due to the surface chlorine accumulation.^[^ ^2^ ^]^ The CB conversion over LMRO decreases by 3.6% in 30 h, lower than that over Ru/LMO (4.4%) and LMO (4.7%), revealing that LMRO retains its activity better and owns superior stability thanks to the existence of Ru and high mobility of surface lattice oxygen, which could facilitate chlorine removal and alleviate the Cl‐poisoning effect. The Ru content of used LMRO and Ru/LMO (0.6% and 0.61%, respectively) is close to that of fresh catalysts, suggesting the stable existence of Ru on the catalysts under the reaction condition.

**Figure 5:**
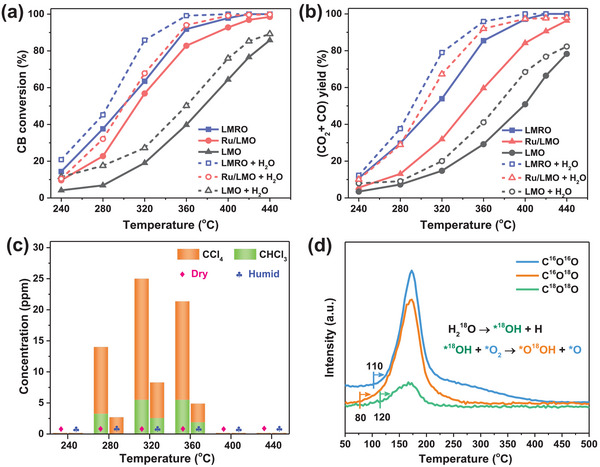
Catalytic behaviors in CB hydrolysis oxidation. a) CB conversion and b) CO*_x_* yield over catalysts in the presence of vapor; c) distribution of chlorinated byproducts over LMRO under dry and humid conditions; d) CO_2_ desorption profiles in CB‐TPSR over LMRO in the presence of H_2_^18^O.

The existence of water vapor is known to have a great influence on CVOCs catalytic oxidation, and the process of hydrolysis oxidation is worthy studying. Accordingly, 3 vol% water vapor is introduced into the feed gas and the catalytic performances of perovskite‐based catalysts are investigated. Both CB conversion and CO*_x_* yield (**Figure** **5**a,b) have been remarkably improved over all catalysts, suggesting that H_2_O could facilitate the destruction of CB into final products. Furthermore, the introduction of H_2_O inhibits the generation of chlorinated byproducts. Only CHCl_3_ and CCl_4_ can be detected during the CB oxidation over LMRO in humid condition, and the concentrations of the produced byproducts are much lower than that in the absence of vapor (Figure 5c). Based on the result of stability test at 350 °C (Figure S12, Supporting Information), LMRO retains a relatively steady CB conversion (above 89%) within 30 h in the presence of H_2_O, exhibiting a superior durability compared to the condition of dry feed gas, and the Ru content is also well remained (0.61%). These results signify that the introduction of H_2_O triggers hydrolysis oxidation of CB, presenting a higher conversion and mineralization rate, less byproducts production and better stability, and the mechanism is discussed in following text.

### 2.4 Investigation of Reaction Mechanism

**Scheme 1:**
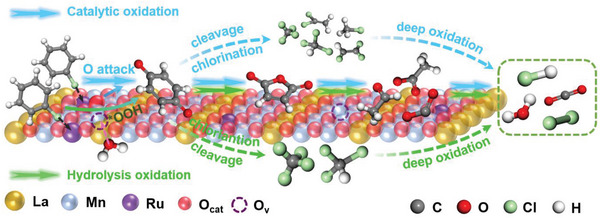
Reaction mechanism of CB oxidation over LMRO

In situ DRIFTS measurements are conducted to probe the reaction mechanism. Under dry condition (Figure S13a, Supporting Information), the bands intensity of OH groups increase with temperature rise, due to the adsorption of generated H_2_O from CB oxidation. The bands at 1603 and 1262 cm^−1^ are attributed to vibration of aromatic ring and C–O stretching vibration of phenolate species, respectively,^[^ ^40^ ^]^ indicating the transformation of adsorbed CB into phenolate. The bands at 1868, 1747, 1688, and 1373 cm^−1^ correspond to maleic anhydride, C=O stretching vibration of aldehyde species, benzoquinone‐type species and bidentate formats, respectively,^[^ ^41^, ^42^, ^43^ ^]^ and the intensity of these intermediates become stronger when raising the temperature. Additionally, the vibration of (chlorinated)‐maleates and acetates are detected at 1458 and 1420 cm^−1^.^[^ ^44^, ^45^ ^]^ Combining in situ DRIFTS analyses with previous reports,^[^ ^29^, ^46^, ^47^ ^]^ the reaction mechanism of CB oxidation can be deduced and shown in **Scheme** **1**. Gaseous CB is first adsorbed at active sites (Ru–O–Mn) with the breakage of C–Cl bond while phenolate species are formed via nucleophilic substitution, which are oxidized into quinines species by active oxygen species followed by the formation of maleic anhydride through ring cleavage. The generated intermediates are decomposed into aldehyde species, acetates and bidentate formates, which are finally oxidized into H_2_O, CO_2_, and HCl/Cl_2_.

After the introduction of H_2_O (Figure S13b, Supporting Information), the bands of hydrogen‐bonded OH and free OH groups getting stronger suggests the boosted dissociated adsorption of H_2_O with temperature increase. Compared with dry condition, the varieties of intermediates barely change whereas the intensity of these bands increase distinctly, confirming that water vapor does not alter the reaction pathways but promotes CB destruction efficiency. In order to explain this promotion, H_2_^18^O is used to label CO_2_ produced in CB/H_2_^18^O‐TPSR (Figure 5d). Three kinds of carbon dioxide containing ^16^O and/or ^18^O are detected, and the initial production temperature follows the order of C^16^O^18^O (80 °C) < C^16^O^16^O (110 °C) < C^18^O^18^O (120 °C), which means H_2_^18^O directly participates in CB oxidation and provides higher‐activity oxygen‐containing species. As reported, H_2_O could be adsorbed on O_v_ or react with surface oxygen to generate active hydroxyl species.^[^ ^48^, ^49^, ^50^, ^51^ ^]^ Combining this with our results, it is deduced that H_2_O is first adsorbed at O_v_ and dissociated into *OH and H, *OH further reacts with O_ads_ to generate *OOH with higher activity than origin O_ads_. Therefore, the process of hydrolysis oxidation enhances the destruction of CB but remains the similar reaction routes as dry condition. Additionally, the oxidation of CB over LMRO with H_2_O pre‐adsorption is performed. The obtained spectra (Figure S14, Supporting Information) show that the formed OH groups after H_2_O adsorption gradually decrease as the reaction proceeds, further confirming these OH groups are converted and participated in the hydrolysis oxidation of CB.

## 3 Conclusion

In summary, for realizing high‐efficiency and safe disposal of CVOCs via catalytic oxidation, a promising Ru‐perovskite catalyst with favorable active sites and optimized Mn–O bond strength is constructed by partial substitution of Ru into LaMnO_3+_ *_*δ*_* bulk. The formed Ru–O–Mn sites facilitate the adsorption and activation of CB molecules, while the weakened Mn–O strength boots the formation of active oxygen species and enhances oxygen mobility and catalyst reducibility. Consequently, LMRO exhibits higher activity of CB destruction and superior capacity of deep oxidation of chlorinated byproducts, accomplishing facile CVOCs degradation, and avoiding second pollution by hazardous byproducts. The higher yield of HCl over LMRO is associated with its surface Brønsted acid sites. Moreover, the introduction of H_2_O triggers hydrolysis oxidation process and generates *OOH species with higher activity, which further promotes CB destruction and inhibits the production of chlorinated byproducts. This study provides a new approach to developing efficient and eco‐friendly catalysts for CVOCs elimination, and inspire the utilization of hydrolysis oxidation technique for optimizing CVOCs degradation process.

## 4 Experimental Section

### Catalysts Synthesis

The pristine LaMnO_3+_ *_*δ*_* perovskite (LMO) was prepared following a simple sol–gel method. The LaMn_0.98_Ru_0.02_O_3+_ *_*δ*_* (LMRO) was synthesized in similar procedure with the partial substitution of Ru for Mn. For comparison, a wet impregnation route was used for the preparation of surface‐loaded Ru/LaMnO_3+_ *_*δ*_* (Ru/LMO). The detailed preparation processes are presented in the Supporting Information.

### Catalysts Characterization and DFT Calculations

The textural and physicochemical properties of the prepared catalysts including crystal phase, specific surface area, morphology, valence states, coordination environment, reducibility, oxygen mobility, CB adsorption capacity, surface acidity, and reaction mechanism were characterized by various techniques. All the density functional theory calculations were performed by the Vienna Ab initio Simulation Package (VASP) with the projector augmented wave (PAW) method.^[^ ^52^, ^53^ ^]^ The exchange‐functional was treated using the generalized gradient approximation (GGA) with Perdew–Burke–Emzerhof (PBE) functional.^[^ ^54^ ^]^ The details of characterizations and DFT computational methods are provided in the Supporting Information.

### Catalytic Performance Measurements

The catalytic activities of obtained perovskite‐based catalysts for CB oxidation were evaluated in a fixed‐bed quartz tube micro‐reactor. The reaction stream was the mixture of 500 ppm CB, 21% O_2_/N_2_ and 3 vol% H_2_O (when used) with a total flow rate of 150 mL min^−1^, giving a gas hourly space velocity (GHSV) of 22 500 mL g^−1^ h^−1^. The details of catalytic performances tests are shown in the Supporting Information.

## Conflict of Interest

The authors declare no conflict of interest.

## Supporting information

Supporting Information

## Data Availability

The data that support the findings of this study are available from the corresponding author upon reasonable request.
