# Supplementary material for: Simultaneously Constructing Active Sites and Regulating Mn–O Strength of Ru‐Substituted Perovskite for Efficient Oxidation and Hydrolysis Oxidation of Chlorobenzene
Source: Adv Sci (Weinh). 2022 Nov 27;10(3):2205054. doi: 10.1002/advs.202205054 (PMC9875690; doi:10.1002/advs.202205054)
Supplement: Supplementary file 1 — Supporting Information [file ADVS-10-2205054-s001.pdf]

ADVANCED SCIENCE
Open Access

Supporting Information

for *Adv. Sci.*, DOI 10.1002/advs.202205054

Simultaneously Constructing Active Sites and Regulating Mn–O Strength of Ru-Substituted Perovskite for Efficient Oxidation and Hydrolysis Oxidation of Chlorobenzene

Xiaoxiao Duan, Ting Zhao, Ben Niu, Zheng Wei, Ganggang Li, Zhongshen Zhang*, Jie Cheng* and Zhengping Hao*

Supporting Information

Simultaneously Constructing Active Sites and Regulating Mn–O Strength of Ru-substituted Perovskite for Efficient Oxidation and Hydrolysis Oxidation of Chlorobenzene

Xiaoxiao Duan, Ting Zhao, Ben Niu, Zheng Wei, Ganggang Li, Zhongshen Zhang*, Jie Cheng* and Zhengping Hao*

National Engineering Laboratory for VOCs Pollution Control Material & Technology
Research Center for Environmental Material and Pollution Control Technology
University of Chinese Academy of Sciences
Beijing, 101408, PR China
E-mail: liganggang@ucas.ac.cn; zszhang@ucas.ac.cn; jiecheng@ucas.ac.cn

Supplementary Experiments

Catalysts Synthesis: For the preparation of $LaMnO_{3+\delta}$ perovskite (LMO), quantitative lanthanum nitrate (0.03 mol) and manganese nitrate (0.03 mol) were dissolved in requisite amount of deionized water under vigorous stirring. Then citric acid and ethylene glycol were added into the homogeneous solution, and the molar ratio of metal cations: citric acid: ethylene glycol is 1: 1.5: 3. Afterwards, the solution was evaporated at 80°C to produce a viscous gel, followed by drying at 100°C overnight. The spongy material, after grinding, was calcined at 200°C for 1 h and 750°C for 4 h with the heating rate of 1°C/min. The similar procedure was used to synthesize $LaMn_{0.98}Ru_{0.02}O_{3+\delta}$ (LMRO) except that aforementioned 0.003 mol manganese nitrate was replaced by 0.0294 mol manganese nitrate and 0.0006 mol ruthenium nitrosyl nitrate. As for the preparation of Ru/$LaMnO_{3+\delta}$ (Ru/LMO), a wet impregnation route was used, where 1g LMO was mixed with 20 mL ruthenium nitrosyl nitrate solution (the concentration is 1.5% w/v Ru) for 2 h, then the suspension was evaporated by a rotary evaporator. Finally, the solid was dried at 80°C followed by calcination at 450°C for 4 h.

Catalysts Characterization and DFT Calculation: X-ray diffraction (XRD) patterns of catalysts were obtained on a Bruker XRD D8 Advance X-ray diffractometer with Cu K□ radiation (40 kV and 40 mA). The practical contents of Ru were measured by inductively coupled plasma-optical emission spectrometer (ICP-OES) technique using an Agilent ICPOES730 spectrometer. The nitrogen adsorption-desorption isotherms were recorded on an ASAP 2020 PLUS HD88 instrument at the temperature of 77 K, and the samples were pretreated at 300°C for 3 h before analysis. The specific surface area was measured by Brunauer-Emmett-Teller (BET) model. Electron paramagnetic resonance (EPR) spectra were obtained on an electron paramagnetic resonance spectrometer (Bruker, E500) at room temperature.

Aberration-corrected high-angle annular dark filed scanning transmission electron microscopy (AC-HAADF-STEM) was performed at 200 kV on a JEOL JEM-ARM 200F electron microscope equipped with an EDS detector. The data of electron energy loss spectroscopy (EELS) was acquired on a matched spectrometer for the analysis of electronic structures and chemical compositions. X-ray photoelectron spectroscopy (XPS) analysis were performed on an AXIS Supra apparatus with Al K□ X-ray source and binding energies were calibrated with the C1s peak at 284.6 eV.

The structures of samples were examined by Raman spectroscopy (Renishaw inVia

confocal Raman) with the use of 532 nm^{-1} laser light. *Ex-situ* Raman spectrum were measured at room temperature. *In-situ* Raman spectrum were measured under different temperatures in an *in-situ* reaction cell. Before the CB oxidation test, the catalyst was pretreated by 21% O_2/N_2 at 400 ℃ for 1 h and then cooled to 200°C. The reactant gas was identical with catalytic performance measurement, which consisted of 500 ppm CB and 21% O_2/N_2. Mn K-edge extended X-ray absorption fine spectra (EXAFS) were performed at 1W1B station in Beijing Synchrotron Radiation Facility (BSRF). The storage rings of BSRF were operated at 2.5 GeV with a maximum current of 250 mA. The Si (111) double-crystal monochromator was used to detect the signal which were carried out in transmission mode in ionization chamber. All spectra were collected in ambient conditions. The EXAFS data were analyzed by the Artemis module of IFEFFIT software packages. First, k^3-weighted EXAFS spectra were obtained by subtracting the post-edge background from the overall absorption and then normalizing with respect to the edge-jump step. Then the obtained data were Fourier transformed to R space to separate the EXAFS contributions from different coordination shells. The quantitative structural parameters around central atoms, least-squares curve parameter fitting was performed using the ARTEMIS module of IFEFFIT software packages.

H_2 temperature-programmed reduction (H_2-TPR) was carried out on a Micromeritics Chemisorb 2920 instrument with a TCD detector. Before each test, the catalyst (50 mg) was pretreated at 400°C for 1 h under the flow of purified Ar (50 mL/min). After cooled down to 50°C, the catalyst was heated to 800°C at the rate of 10°C/min in a stream of 10% H_2/Ar (50 mL/min). For O_2 temperature-programmed desorption (O_2-TPD) experiments, the catalyst was first pretreated at 400°C in He flow for 1 h followed by being cooled to 50°C. Then the catalyst was exposed to the stream of 5% O_2/He (50 mL/min) for 30 min and purged by pure He afterwards for the removal of residual gaseous O_2. Lastly, the temperature was increased to 850°C at the rate of 10°C/min under a He flow and the signal of desorbed O_2 was recorded by a mass spectrometer (HIDEN, HPR-20 R&D).

For temperature-programmed surface reaction of CB (CB-TPSR), the sample was first exposed in the flow of 21% O_2/N_2 at 400°C for 1 h and cooled to 50°C, at which temperature the adsorption of CB and $H_2{}^{18}O$ (when used in CB/$H_2{}^{18}O$-TPSR) was carried out. After saturation, the sample was purged by helium for the removal of physically adsorbed CB on the surface. Then the sample was heated to 900°C in a flow of 5% O_2/He while the MS signal of CO_2 and O_2 were recorded. Temperature-programmed desorption of CB (CB-TPD) was performed with similar procedure, except that a flow of pure He was used when temperature was risen up to 900°C and CB signal was recorded.

Pyridine-IR spectra were collected by using a Bruker Tensor II spectrometer equipped with a quartz cell connected to a vacuum system. A thin wafer was obtained through crushing the powder catalyst and pretreated at 350°C for 1 h under vacuum. After cooled down to room temperature, the catalyst was connected to pyridine for adsorption (30 min). Then the desorption was carried out under vacuum with temperature rising to 100°C, and the spectra were recorded at 30 and 100°C.

For *in situ* diffuse reflectance infrared Fourier transform spectroscopy (DRIFTS), the experiments were conducted in a chamber with BaF_2 windows and the catalysts could be heated to 550°C. Prior to CB oxidation, the catalysts were pretreated at 400°C for 1 h under a flow of nitrogen followed by being cooled to 100°C and the spectra were recorded as background. Then the catalysts were exposed to mixed gas stream (60 mL/min) of 500 ppm CB, 21% O_2/N_2 and 3 vol. % (when used), meanwhile, the sample chamber was heated and DRIFTS spectra were collected at various temperatures.

As for DFT calculations, the energy cutoff for the plane wave basis expansion was set to 400 eV. Partial occupancies of the Kohn-Sham orbitals were allowed using the Gaussian smearing method and a width of 0.2 eV. The Brillourin zone was sampled with Monkhorst mesh of 4×4×4 for the optimization for the bulk structure of LMO. According to the experimental results, bare LMO (110) surface was built first, and a cluster of RuO_2 was considered to be loaded on the surface of LMO (110), which was named as Ru/LMO (110). In addition, Mn was also considered to be replaced by single atom Ru on LMO (021) surface. The Monkhorst mesh of 2×2×1 was used in all the surface structure calculations. The energy convergence was set to 10^{-5} eV, and the force convergency was set to 0.05 eV/Å.

Catalytic Performance Measurements: Equivalent amount of catalyst (0.4 g) was used for each test of catalytic activity. An Agilent 6890 gas chromatograph equipped with a flame ionization detector (FID) was used to monitor the concentrations of CB and other chlorinated organics. CO, CO_2 and HCl were analyzed by an on-line FTIR spectrometer (MKS, MultiGas 2030). The conversion of CB and yields of CO_2, CO and HCl were calculated on the basis of following equations:

$$X=\frac{[CB]_{in}-[CB]_{out}}{[CB]_{in}}\times 100\% \quad (1)$$

$$Y_{CO_2}=\frac{[CO_2]_{out}}{6[CB]_{in}}\times 100\% \quad (2)$$

$$Y_{CO}=\frac{[CO]_{out}}{6[CB]_{in}}\times 100\% \quad (3)$$

$$Y_{CO_x}=\frac{[CO_2]_{out}+[CO]_{out}}{6[CB]_{in}}\times 100\% \quad (4)$$

$$Y_{HCl}=\frac{[HCl]_{out}}{[CB]_{in}}\times 100\% \quad (5)$$

where $[CB]_{in}$ and $[CB]_{out}$ represents the CB concentrations in inlet and outlet gas stream, and $[CO]_{out}$, $[CO_2]_{out}$ and $[HCl]_{out}$ represents the concentrations of CO, CO_2 and HCl in outlet gas, respectively.

For the calculation of apparent activation energies of catalysts, the reaction rates of each sample show negligible change under the GHSV at 11000 $mL\cdot g^{-1}\cdot h^{-1}$, 22500 $mL\cdot g^{-1}\cdot h^{-1}$ and 45000 $mL\cdot g^{-1}\cdot h^{-1}$, indicating that the effect of external diffusion could be eliminated by using GHSV regulation. The particle size of catalysts between 40-60 mesh was chosen for the elimination of internal diffusion.[1] The following Arrhenius equation was used and the CB conversion was controlled to lower than 15%.

$$\ln r = -\frac{E_a}{RT} + \ln A \quad (6)$$

$$r = \frac{F \times X}{W} \quad (7)$$

where r is the CB reaction rate ($mol\cdot s^{-1}\cdot g_{cat.}^{-1}$), E_a is the apparent activation energy ($kJ\cdot mol^{-1}$), R is the molar gas constant (8.314 $J\cdot mol^{-1}\cdot K^{-1}$), T is the reaction temperature (K), A is the pre-exponential factor. F represents the flow rate of CB (mol/s), X is the CB conversion and W is the amount of each catalyst (g).

Supplementary Figures

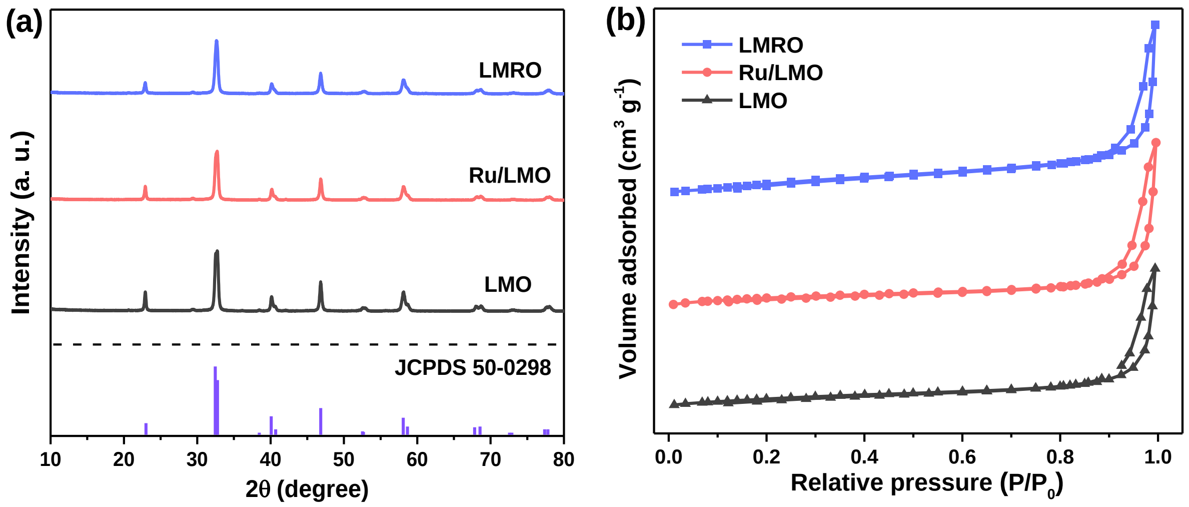

Figure S1. (a) XRD patterns and (b) N_2 adsorption-desorption isotherms of LMRO, Ru/LMO and LMO.

Note: The N_2 adsorption-desorption isotherms proves the mesoporous structure of these perovskite-based catalysts.[2]

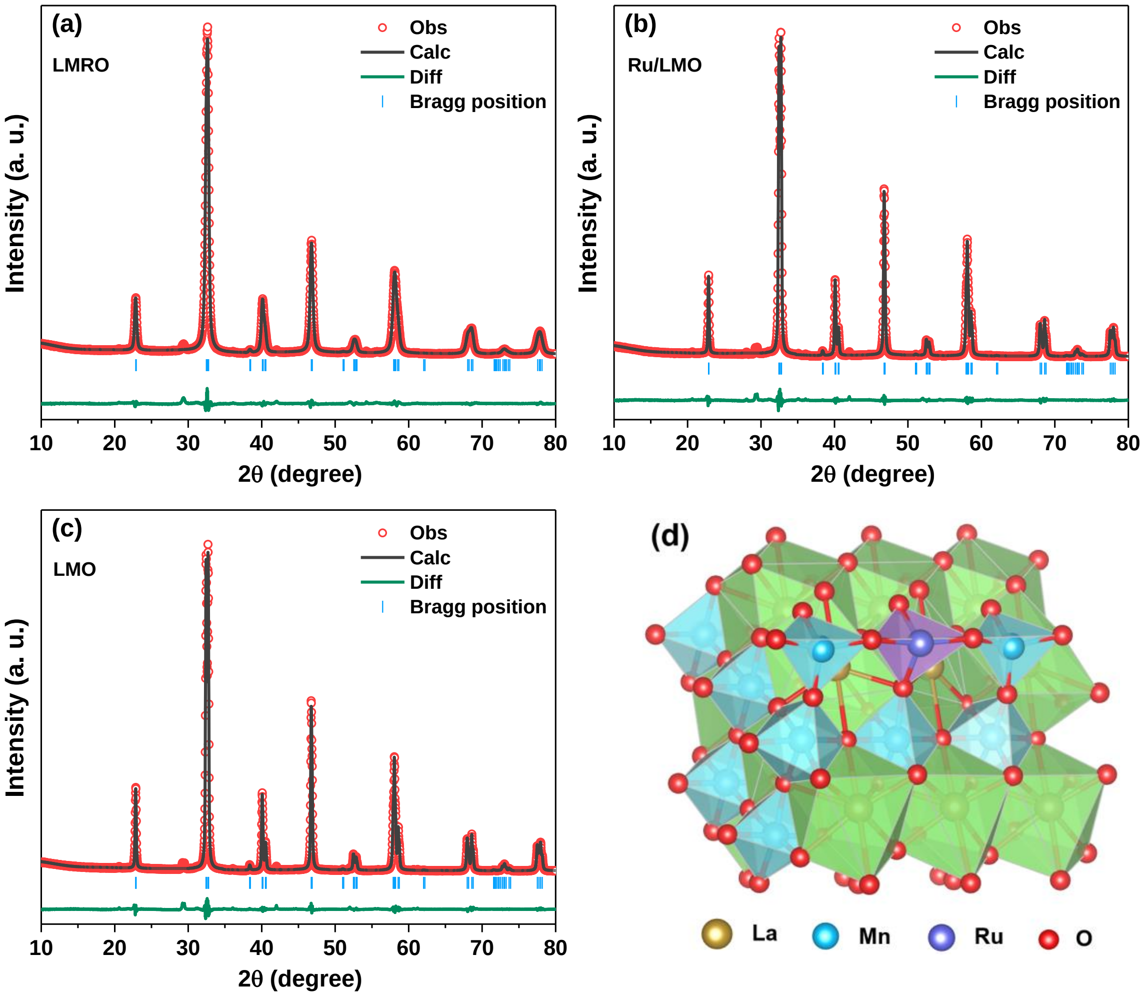

Figure S2. Rietveld refined XRD patterns of (a) LMRO, (b) Ru/LMO and (c) LMO, (d) simulated crystal structure of LMRO.

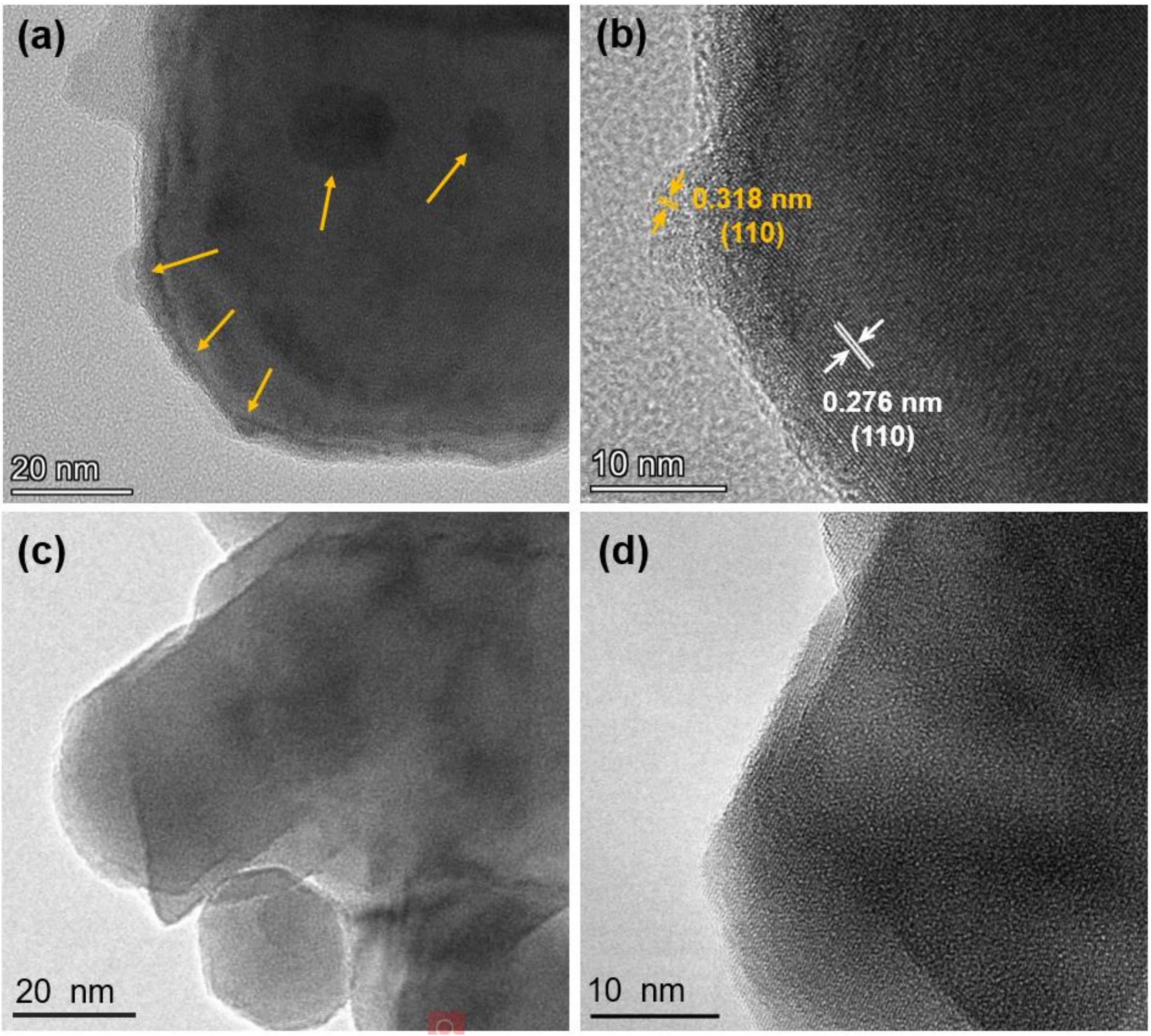

Figure S3. TEM images of (a) (b) Ru/LMO and (c) (d) LMRO.

Note: In Figure S3, the TEM images of Ru/LMO (Figure S3a and b) show that RuO_x nanoparticles (pointed by arrows) are supported on the surface of perovskite with preferentially exposed (110) plane. According to the lattice spacing of 0.318 nm of RuO_x ((110) plane of RuO_2), the chemical form of surface-loaded Ru is RuO_2. No obvious RuO_x nanoparticles can be found on the surface of LMRO (Figure S3c and d).

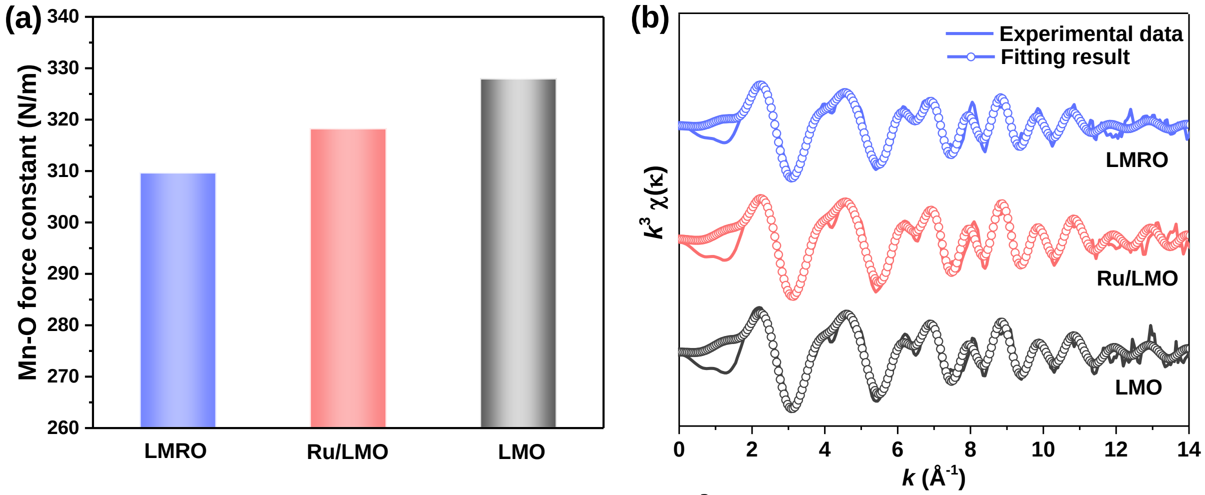

Figure S4. (a) Mn–O bond force constant and (b) k^3-weighted EXAFS oscillations of as-obtained catalysts.

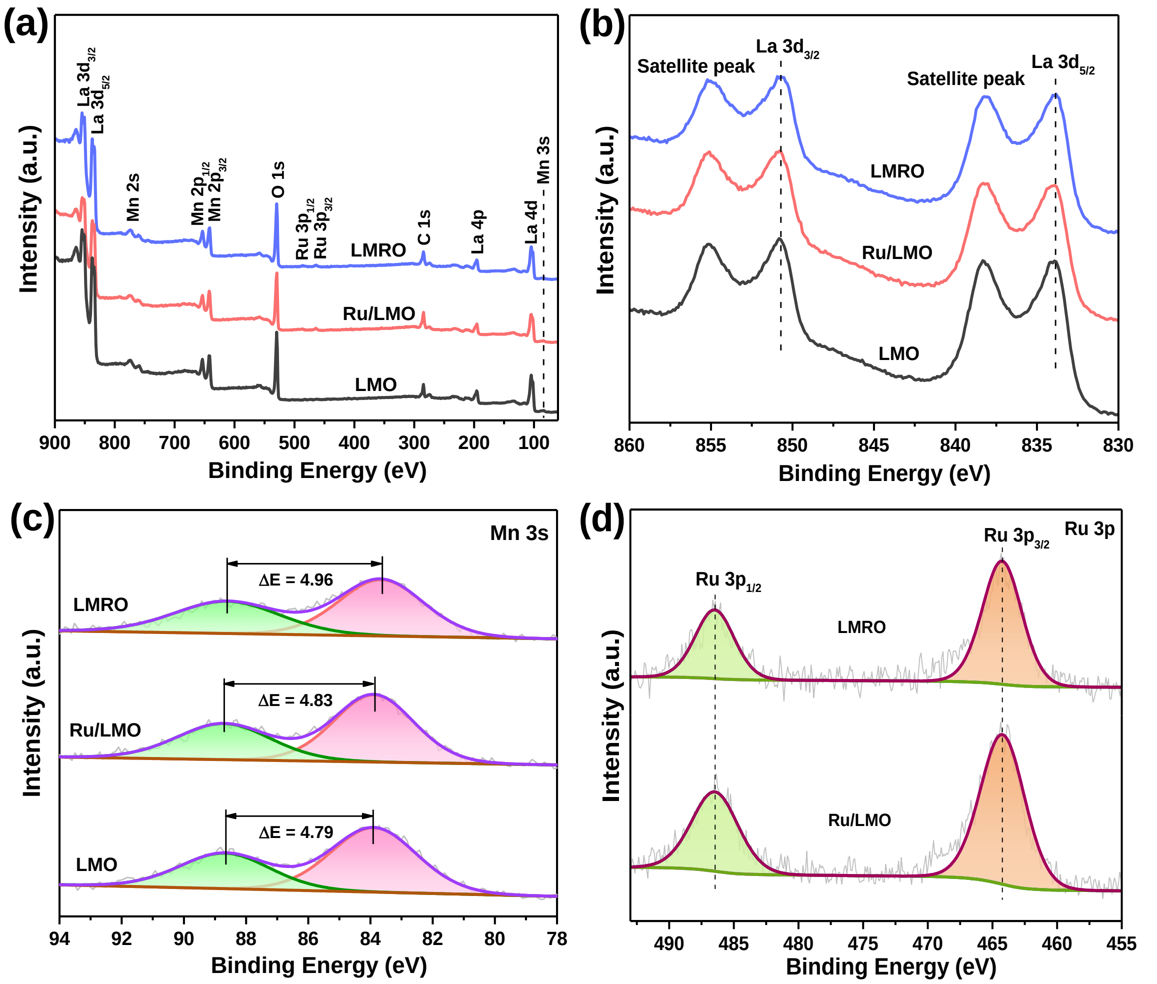

Figure S5. (a) XPS survey, and (b) La 3d, (c) Mn 3s and (d) Ru 3p XPS spectra of the prepared catalysts.

Note: Figure S5a provides the XPS survey of all catalysts. The La 3d XPS spectra in Figure S5b contain two main peaks at 834.1 and 850.8 eV, corresponding to La $3d_{5/2}$ and La $3d_{3/2}$, respectively. Another two peaks at 838.3 and 855.2 are satellite peaks of La $3d_{5/2}$ and La $3d_{3/2}$.[3] It can be found that the chemical states of La in the catalysts have little change, indicating that the element La is not directly related to the change of catalytic activity.

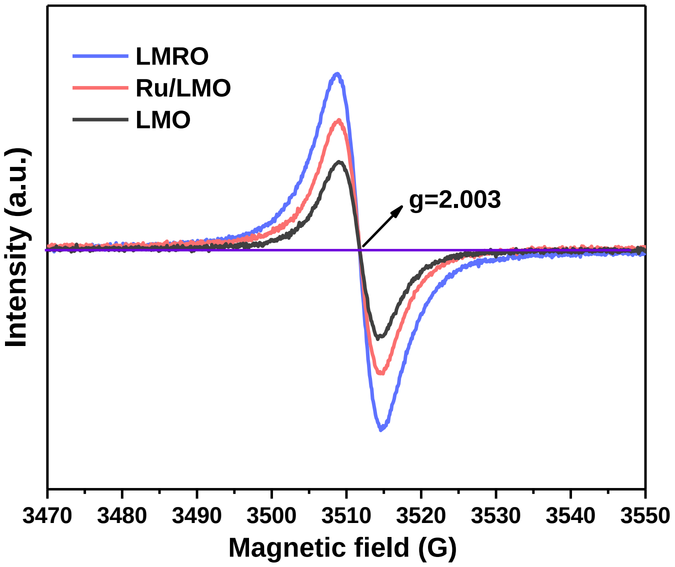

Figure S6. EPR profiles of as-prepared catalysts.

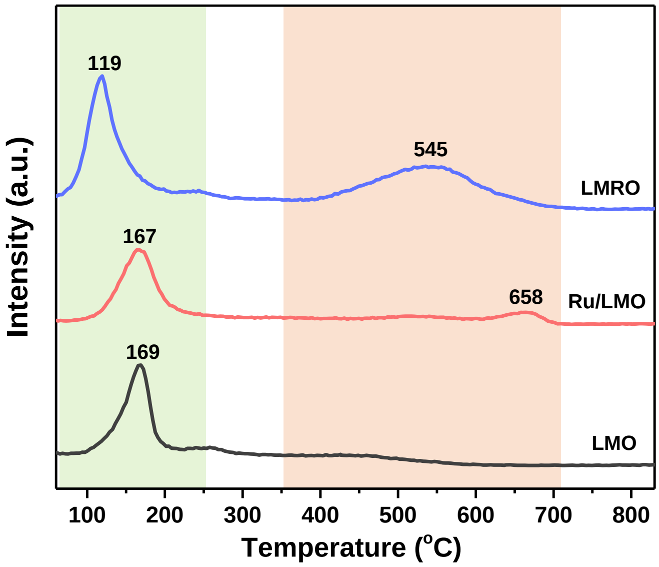

Figure S7. CO_2 production profiles during CB-TPSR over the perovskite-based catalysts.

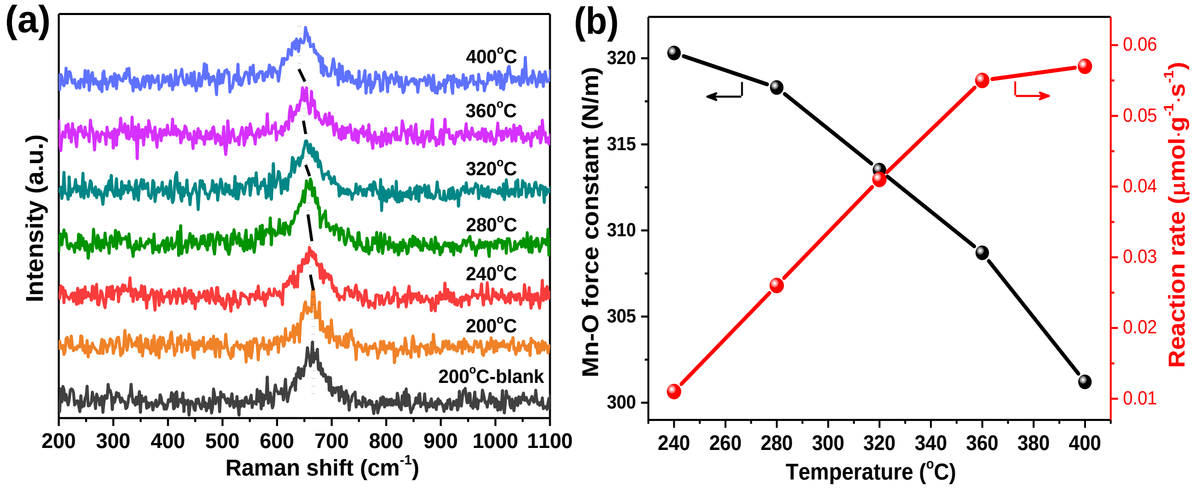

Figure S8. (a) in situ Raman spectra and (b) Mn–O bond force constant of LMRO and reaction rate at various temperatures.

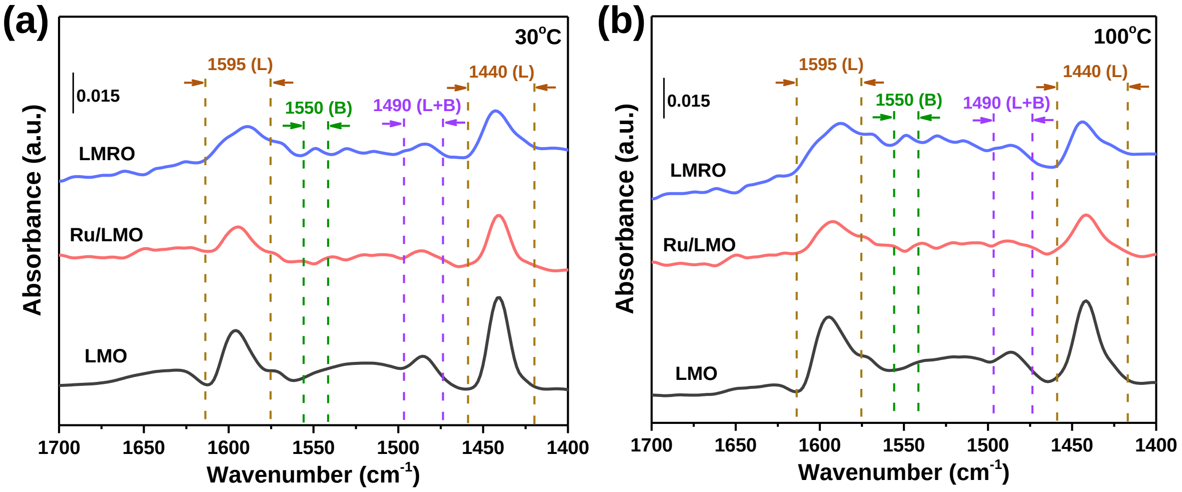

Figure S9. Pyridine-IR spectra of LMO, Ru/LMO and LMRO recorded at (a) 30°C and (b) 100°C.

Note: In Figure S8, the IR bands at about 1440 and 1595 cm^{-1} are assigned to pyridine adsorbed at Lewis acid sites, while the peak at 1550 cm^{-1} should be related to Brønsted acid sites.[4,5] Another band corresponding to both Lewis acid sites and Brønsted acid sites is observed at 1490 cm^{-1}.[6] The intensity of adsorption peaks gets weakened slightly when the temperature rises up, which indicates the relative strong strength of acidity on these catalysts.

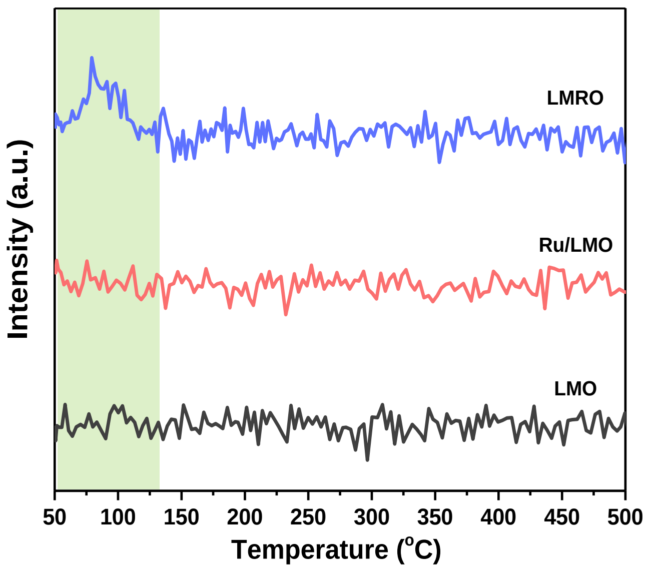

Figure S10. CB desorption profiles over the obtained catalysts in CB-TPD.

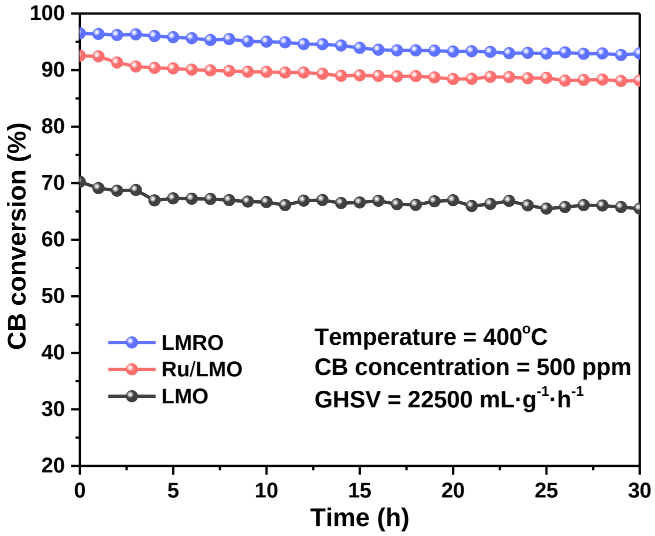

Figure S11. Stability of the prepared catalysts for CB oxidation at 400°C.

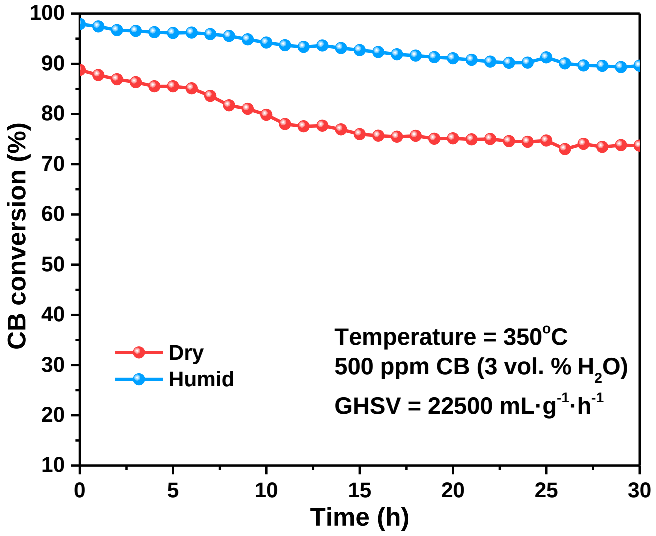

Figure S12. Stability of LMRO for CB oxidation at 350°C under dry and humid conditions.

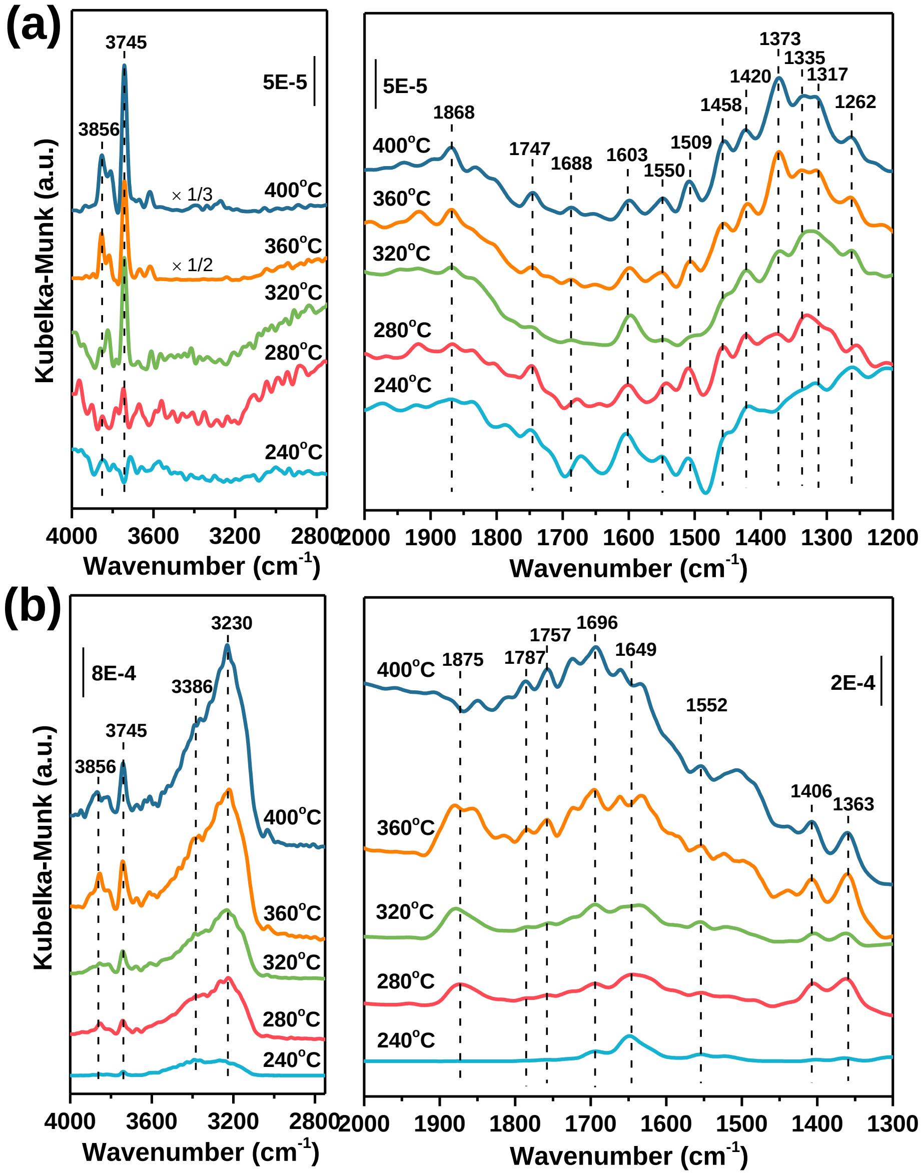

Figure S13. In situ DRIFTS spectra of CB catalytic oxidation over LMRO in the (a) absence and (b) presence of water vapor at various temperatures.

Note: In Figure S12a, the bands at 3856 and 3745 cm^{-1} should be assigned to free OH groups of H_2O,[7] the intensity of which increase with temperature rising. The phenolate species (1603 and 1262 cm^{-1}) on the surface come from the CB adsorption via cleavage of C–Cl bond.[8] The vibration peak at 1550 cm^{-1} is due to carboxylate[9,10] while the bands at 1509, 1458 and 1420 cm^{-1} are assigned to COO- symmetric and antisymmetric stretching vibration of (chlorinated)-maleates and acetates.[11-13] Besides, the bands at 1373, 1335 and 1317 cm^{-1} can be ascribed to –COOH from bidentate formats.[14] In Figure S12b, the bands at 3386, 3230 and 1649 cm^{-1} are attributed to hydrogen-bonded OH groups,[15] the bands of which increase in intensity with the rise of temperature. The bands at 1875 and 1696 cm^{-1} are due to

the vibration of maleic anhydride and aldehyde species, respectively. The intensity of these two bands gets obviously stronger under humid condition.

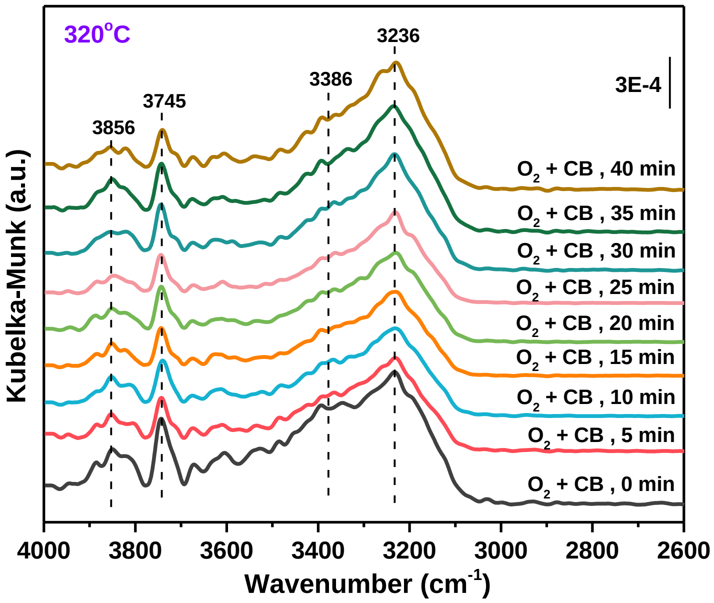

Figure S14. In situ DRIFTS spectra of CB catalytic oxidation over LMRO with water vapor pre-adsorption.

Note: After the adsorption of H_2O, the bands attributed to free OH groups and hydrogen-bonded OH groups can be observed. The intensities of these bands gradually decrease with the extension of reaction time within 20 minutes, and then get stronger after 25 minutes because of the produced H_2O from reaction.

Supplementary Tables

Table S1. Textural properties of LMRO, Ru/LMO and LMO.

Catalyst	Ru content[a]	Crystallite size (nm)[b]	Lattice parameter (nm)[c]			S_{BET}[d] ($m^2 \cdot g^{-1}$)
			a	*b*	*c*	
LMRO	0.61%	27.8	0.5505	0.5505	1.3355	27.5
Ru/LMO	0.62%	29.9	0.5506	0.5506	1.3332	20.4
LMO	–	31.1	0.5509	0.5509	1.3333	21.1

[a] Measured by ICP-OES.

[b] Calculated by Scherrer equation from XRD patterns.

[c] Obtained from Rietveld refined XRD patterns.

[d] Measured by N_2 adsorption with BET model.

Table S2. EXAFS fitting parameters at Mn K-edge for prepared catalysts.

Catalyst	Shell	R[a] (Å)	[b]σ^2 ($Å^2 \cdot 10^{-3}$)	ΔE_0[c] (eV)	R factor (%)
LMRO	Mn–O	1.96	0.0072	3.08	1.2
Ru/LMO	Mn–O	1.94	0.0053	1.52	1.5
LMO	Mn–O	1.91	0.0060	3.59	1.3

Note: [a] R is the Mn–O distance, [b] σ^2 is Debye-Waller factor to account for thermal and structural disorders, [c] ΔE_0 is inner potential correction, R factor indicates the goodness of the fit, and S_0^2 is fixed to 0.71.

Table S3. XPS results and catalytic activity of prepared catalysts.

Catalyst	Mn^{3+}/Mn^{4+}	AOS of Mn[a]	O_{ads}/O_{latt}	Catalytic activity		E_a (kJ/mol)
				T_{50} (°C)	T_{90} (°C)	
LMRO	5.09	3.37	0.88	298	356	43.5
Ru/LMO	3.59	3.52	0.78	312	388	53.9
LMO	2.72	3.56	0.64	376	>440	86.3

Note: [a] AOS of Mn is calculated according to the equation: AOS = 8.956-1.126ΔE_{3s},[16] where ΔE_{3s} is the binding energy gap between two main peaks of Mn 3s spectra.

Table S4. Acidic properties of as-prepared catalysts measured from Pyridine-IR.

Catalyst	Lewis acidity[a] (μmol/$g_{cat.}$)		Brønsted acidity[b] (μmol/$g_{cat.}$)		Total acidity (μmol/$g_{cat.}$)	
	30°C	100 °C	30°C	100°C	30°C	100°C
LMRO	29.30	28.10	2.38	2.12	31.68	30.22
Ru/LMO	30.90	29.10	1.19	0.93	32.09	30.02
LMO	50.90	48.90	–	–	50.90	48.90

Note: [a] Lewis acidity = 1.42 × IA(L) × R^2/W, [b] Brønsted acidity = 1.88 × IA(B) × R^2/W, where IA(L) and IA(B) represent integrated absorbance of Lewis acid bond and Brønsted acid band, respectively, R is the radius of catalyst disk (cm), and W is the weight of disk (mg).[17]

Table S5. Catalytic performance comparison of LMRO with other reported catalysts.

Catalyst	Gas composition	GHSV mL/(g·h)	T_{50} (°C)	T_{90} (°C)	Ref.
LMRO	500 ppm CB, 21% O_2, N_2	22500	298	356	This work
Mn/Al_2O_3	100 ppm CB, 20% O_2, N_2	40000 h^{-1}	345	478	[18]
$S\text{-}Ce_{0.7}Zr_{0.3}O_2$	1000 ppm CB, 20% O_2, N_2	60000	346	406	[19]
W/CeO_2	1000 ppm CB, 10% O_2, N_2	60000 h^{-1}	325	387	[20]
Mn-Ce-Zr	1000 ppm CB, 21% O_2, N_2	30000 h^{-1}	326	385	[21]
$Pd/LaCoO_3$	1000 ppm CB, 21% O_2, N_2	12000	342	430	[22]

References

[1] X. Weng, P. Sun, Y. Long, Q. Meng, Z. Wu, *Environ. Sci. Technol.* **2017**, 51, 8057.
[2] F. Zhang, X. Zhang, G. Jiang, Y. Sun, Z. Hao, X. Liu, S. Qu, *Catal. Today* **2020**, 355, 366.
[3] K. A. Ali, A. Z. Abdullah, A. R. Mohamed, *Appl. Catal. A: Gen.* **2017**, 537, 111.
[4] Z. Zhang, H. Xia, Q. Dai, X. Wang, *Appl. Catal. A: Gen.* **2018**, 557, 108.
[5] M. Tian, X. Guo, R. Dong, Z. Guo, J. Shi, Y. Yu, M. Cheng, R. Albilali, C. He, *Appl. Catal. B: Environ.* **2019**, 259, 118018.
[6] Y. Xu, X. Wu, L. Cao, Y. Ma, R. Ran, Z. Si, D. Weng, Z. Ma, B. Wang, *J. Catal.* **2019**, 375, 294.
[7] X. Yu, L. Dai, J. Deng, Y. Liu, L. Jing, X. Zhang, R. Gao, Z. Hou, L. Wei, H. Dai, *Appl. Catal. B: Environ.* **2022**, 305, 121037.
[8] S. L. Alderman, G. R. Farquar, E. D. Poliakoff, B. Dellinger, *Environ. Sci. Technol.* **2005**, 39, 7396.
[9] W. L. Wang, Q. Meng, Y. Xue, X. Weng, P. Sun, Z. Wu, *J. Catal.* **2018**, 366, 213.
[10] G. Wang, Y. Wang, L. Qin, B. Zhao, L. Guo, J. Han, *Catal. Sci. Technol.* **2020**, 10, 7203.
[11] H. Huang, Y. Gu, J. Zhao, X. Wang, *J. Catal.* **2015**, 326, 54.
[12] J. Lichtenberger, M. D. Amiridis, *J. Catal.* **2004**, 223, 296.
[13] Z. Zhang, J. Huang, H. Xia, Q. Dai, Y. Gu, Y. Lao, X. Wang, *J. Catal.* **2018**, 360, 277.
[14] P. Sun, W. Wang, X. Weng, X. Dai, Z. Wu, *Environ. Sci. Technol.* **2018**, 52, 6438.
[15] C. Ma, S. Sun, H. Lu, Z. Hao, C. Yang, B. Wang, C. Chen, M. Song, *J. Hazard. Mater.* **2021**, 414, 125542.
[16] G. Jiang, F. Zhang, Z. Wei, Z. Wang, Y. Sun, Y. Zhang, C. Lin, X. Zhang, Z. Hao, *Catal. Sci. Technol.* **2020**, 10, 1477.
[17] H. Yang, C. Ma, X. Zhang, Y. Li, J. Cheng, Z. Hao, *ACS Catal.* **2018**, 8, 1248.
[18] F. Lin, Z. Wang, Z. Zhang, L. Xiang, D. Yuan, B. Yan, Z. Wang, G. Chen, *Environ. Sci. Technol.* **2021**, 55, 3341.
[19] X. Lv, S. Cai, J. Chen, D. Yan, M. Jiang, J. Chen, H. Jia, *Catal. Sci. Technol.* **2021**, 11, 4581.
[20] Y. Gu, T. Cai, X. Gao, H. Xia, W. Sun, J. Zhao, Q. Dai, X. Wang, *Appl. Catal. B: Environ.* **2019**, 248, 264.
[21] G. Long, M. Chen, Y. Li, J. Ding, R. Sun, Y. Zhou, X. Huang, G. Han, W. Zhao, *Chem. Eng. J.* **2019**, 360, 964.
[22] J. M. Giraudon, A. Elhachimi, G. Leclercq, *Appl. Catal. B: Environ.* **2008**, 84, 251.
